# Intravital Placenta Imaging Reveals Microcirculatory Dynamics Impact on Sequestration and Phagocytosis of *Plasmodium*-Infected Erythrocytes

**DOI:** 10.1371/journal.ppat.1003154

**Published:** 2013-01-31

**Authors:** Luciana Vieira de Moraes, Carlos Eduardo Tadokoro, Iván Gómez-Conde, David N. Olivieri, Carlos Penha-Gonçalves

**Affiliations:** 1 Instituto Gulbenkian de Ciência, Oeiras, Portugal; 2 Escuela Superior de Ingeniería Informatica, University of Vigo, Vigo, Spain; Case Western Reserve University, United States of America

## Abstract

Malaria in pregnancy is exquisitely aggressive, causing a range of adverse maternal and fetal outcomes prominently linked to *Plasmodium*-infected erythrocyte cytoadherence to fetal trophoblast. To elucidate the physiopathology of infected erythrocytes (IE) sequestration in the placenta we devised an experimental system for intravital placental examination of *P. berghei*-infected mice. BALB/c females were mated to C57Bl/6 CFP+ male mice and infected with GFP+ *P. berghei* IE, and at gestational day 18, placentas were exposed for time-lapse imaging acquisition under two-photon microscopy. Real-time images and quantitative measurements revealed that trophoblast conformational changes transiently restrain blood flow in the mouse placental labyrinth. The complex dynamics of placental microcirculation promotes IE accumulation in maternal blood spaces with low blood flow and allows the establishment of stable IE-trophoblast contacts. Further, we show that the fate of sequestered IE includes engulfment by both macrophagic and trophoblastic fetal-derived cells. These findings reinforce the current paradigm that IE interact with the trophoblast and provide definitive evidence on two novel pathogenesis mechanisms: (1) trophoblast layer controls placental microcirculation promoting IE sequestration; and (2) fetal-derived placental cells engulf sequestered IE.

## Author Summary

Malaria in pregnancy is exquisitely aggressive, causing a range of adverse effects impacting maternal and fetal health. Many of those effects are thought to derive from placental sequestration of red blood cells infected with the malaria parasite (*Plasmodium falciparum*) eliciting a placental inflammatory response that impairs maternal-fetal exchanges. We developed an experimental system for intravital microscopy to directly observe the course of placental infection in a mouse model of pregnancy-associated malaria. We found that microcirculation in infected placentas showed areas of low blood flow that promote sequestration of infected red blood cells. Furthermore, we observed that sequestered infected red blood cells are targeted and phagocytosed by fetal-derived cells in the materno-fetal interface. This work provides the first ever *in vivo* evidence that unique placental microcirculatory features promote infected red blood cell sequestration, implying a vascular component in placental malaria pathogenesis. Moreover, we reinforce the notion that fetal-derived cells contribute to the placental response against sequestered infected red blood cells.

## Introduction

Infection with *Plasmodium* parasites during pregnancy is one of the leading causes of maternal and perinatal morbidity and mortality in malaria endemic areas and is particularly severe in regions of unstable transmission [1]. Women infected with *Plasmodium falciparum* experience a range of adverse pregnancy outcomes including abortions, stillbirths, premature delivery and low infant birth weight. Early descriptions of marked accumulation of infected erythrocytes (IE) in the placental intervillous spaces [2] are currently explained by the cytoadherence of *P. falciparum*-infected erythrocytes to low-sulfated chondroitin 4-sulfate (C4S or CSA) proteoglycan present predominantly in the intervillous space of the placenta [3] and on the syncytiotrophoblast lining [4]. Binding of infected cells to placental C4S proteoglycan requires interaction of *P. falciparum* erythrocyte membrane protein 1 (PfEMP1) molecule, namely VAR2CSA expressed on the surface of IE [5], [6]. The current placental malaria (PM) pathogenesis paradigm stipulates that accumulation of IE in human placenta elicits an inflammatory response [7]–[9] that is presumably responsible for pathological changes observed in the placental barrier (interhaemal membrane), which ultimately has a negative impact on fetal growth and viability [10]–[12]. To a large extent, this pathogenesis model is based on seminal findings that correlate human placental pathology with *in vitro* IE adhesion properties [13]. It is still unclear whether placental microcirculation contributes to establishment of *in vivo* IE-throphoblast interactions. IE cytoadherence in the placenta is seen as a strategy of the parasite to circumvent host immunity and propagate the blood stage infection. Little is known of the fate of sequestered IE and the role of fetal components in the subsequent inflammatory response.

Although mouse PM develops within a narrow time window of mouse pregnancy with reduced accumulation of *P.berghei-*IE as compared to human acute PM, pathological findings suggest that the disease involves a strong inflammatory response leading to extensive placental tissue remodeling [14], [15]. *P.berghei-*IE display placental tissue adhesion that is partially dependent on placental extracellular C4S [14], but it is uncertain whether placental sequestration in the mouse is dependent on IE surface antigens mimicking the var2CSA adhesion mechanisms. Observations in experimental mouse models of severe malaria might not have direct correlation to human disease [16]. It should be noted that mouse placental tissue organization and vasculature architecture show marked differences compared to the human placenta [17]. In addition, the exposure time to the parasite is reduced to a few days, conditioning the extent of inflammatory infiltration and immune response observed in mouse PM. Nevertheless, this experimental PM model provides an opportunity to evaluate *in vivo* placenta-IE interactions and track the fate of IE in the placenta, a research that is not amenable in the human placenta.

We used intravital microscopy methods to visualize IE in the placental maternal blood space and analyze the dynamics of IE-placenta interactions. We observed that unexpected maternal blood flow dynamics in the placenta labyrinth favor stable contacts of IE with the fetal trophoblast layers and promote phagocytosis of IE by the fetal cells in the placenta.

## Results

### Experimental model

**Figure 1:**
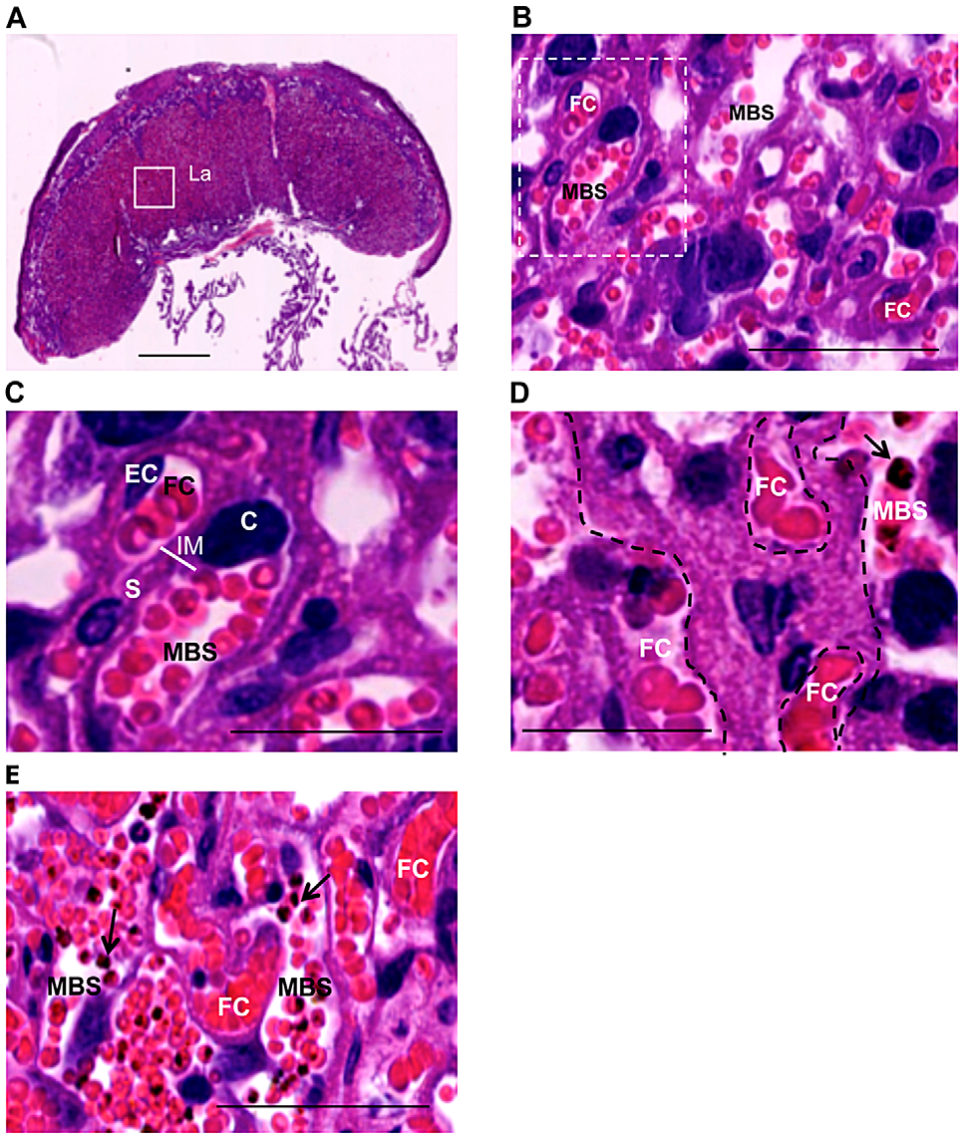
Labyrinth structure in non-infected (A–C) and infected placenta (D,E) at G18. Infection was performed with *P. berghei-*ANKA on G13. (**A**) Sagittal section of the placenta highlighting the labyrinth (La); (**B,C**) Magnified area of the labyrinth of non-infected placenta showing maternal blood space (MBS), fetal capillary (FC) identified by the presence of endothelial cells (EC), cytotrophoblast (C) and syncytiotrophoblast (S) which together form the interhaemal membrane (IM) as depicted in (**C**); (**D**) Thickening of trophoblast layer (delimited area) between maternal and fetal circulations in infected placenta (arrow point to IE); (**E**) Infected erythrocytes (arrows) are restricted to maternal compartment. MBS: maternal blood space; FC: fetal capillary. Scale bars: 1 mm (**A**); 50 µm (**B,E**); 25 µm (**C,D**).

The mouse model of placental malaria is based on infection of pregnant mice at gestational day (G)13 with *P. berghei* ANKA [14]. Placenta observations were performed on G18 in the labyrinth – the inner region of the placenta composed of fetal vasculature and fetal-derived trophoblast layers, the cytotrophoblast and syncytiotrophoblast (Figures 1A and 1B). Together with fetal endothelia, the trophoblast layers in the labyrinth provide a trichorial barrier between maternal and fetal blood circulation, the interhaemal membrane (IM) (Figure 1C) through which nutrients and oxygen are transferred both by diffusion and active transport [18]. In this model, IM thickening is a prominent consequence of *P. berghei* infection during pregnancy [14], [19] (Figure 1D) and congenital infection does not occur, as infected erythrocytes (IE) are restricted to maternal circulation and were never observed in fetal blood vessels [14] (Figure 1E).

To discriminate fetal placental tissue, infected erythrocytes and maternal blood in the infected placenta with fluorescence microscopy we combined three separate tags: i) a label for fetal-derived tissue by crossing BALB/c females with actin-CFP C57Bl/6 (B6.Cyan) males; ii) infection of pregnant mice with *P. berghei* GFP labeled parasites; iii) a label for the maternal blood fluid by Dextran-Rhodamine injection (when applicable) (Figure S1). Using this experimental system we performed intravital placental imaging by means of a novel two-photon microscopy staging technique that allowed visualization of the placental labyrinth displaying CFP^+^ placental components of fetal origin (except for fetal erythrocytes that were CFP^−^), GFP^+^ IE and Rhodamine^+^ maternal blood spaces.

### Trophoblast conditions labyrinth blood flow

**Figure 2:**
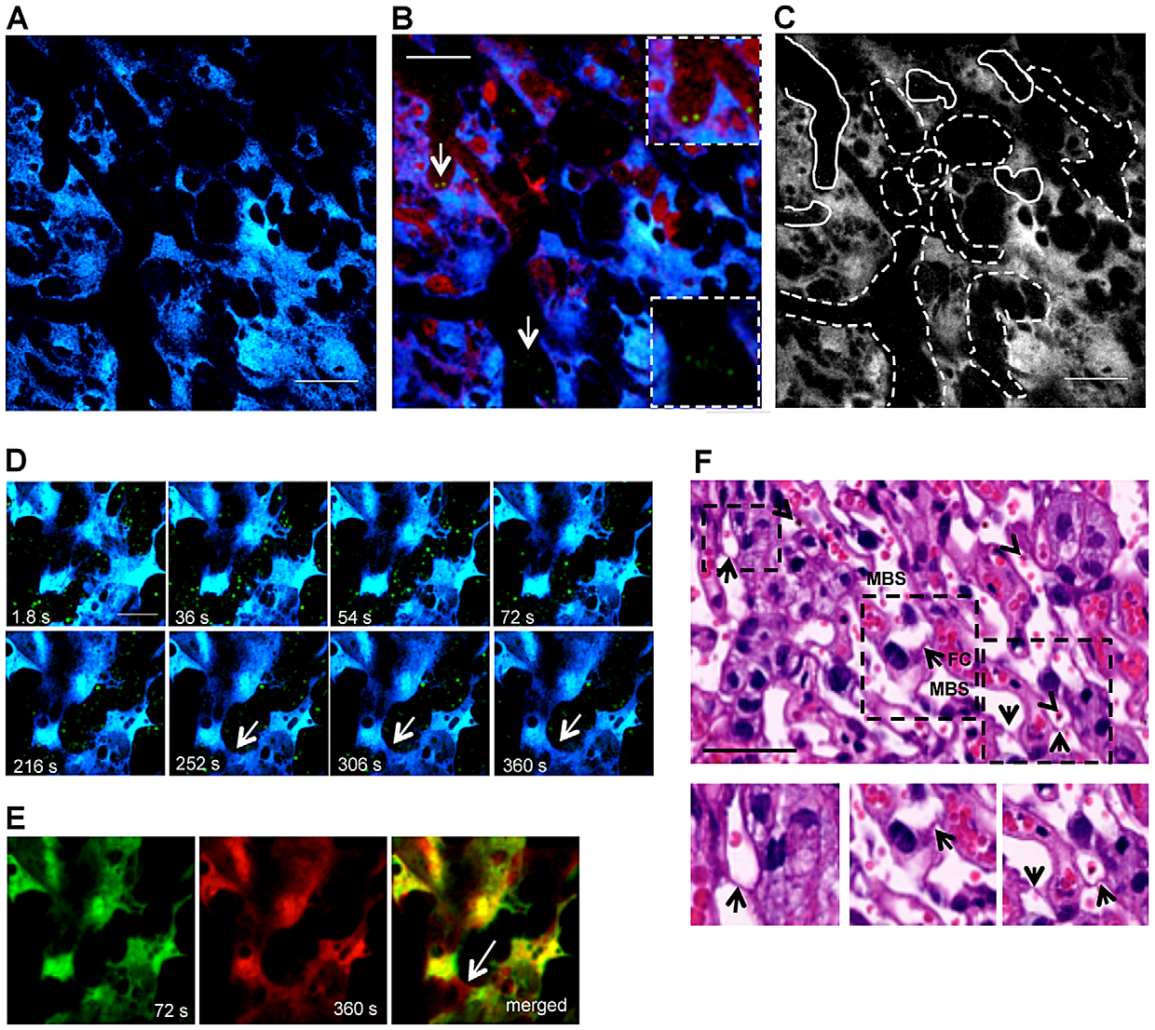
Blood flow rate in the labyrinth of infected placenta is conditioned by trophoblast conformational changes. (**A**) Two-photon intravital image showing fetal-derived CFP^+^ placental tissue (blue) at G18 under transversal incidence after infection with *P. berghei* ANKA-GFP^+^; (**B**) Merged image of GFP, CFP and Rhodamine fluorescence from intravital acquisition (200×) display IE (green; arrows; magnified in the insets) labelled blood fluid (red) in maternal blood space and placental tissue (blue) and (**C**) delimitation of areas with high blood flow (solid lines) or low flow/unperfused (dashed lines) as revealed by a 3 min. observation (see Video S1); (**D**) Sequential images of a 6 min intravital acquisition in higher magnification (600×) in the GFP (green) and CFP (blue) channels showing trophoblast conformational changes over time (see Video S2). Arrow indicates emerging trophoblast bridge; (**E**) Merged image of two time-points (72 seconds colored in green and 360 seconds colored in red) show strong co-localization of areas from both images except for trophoblast bridging structure (arrow); (**F**) H&E histological section of placental labyrinth at G18, on day 5 after infection. Different areas of the labyrinth (dashed squares) are magnified to evidence “Coan-Burton bridges” (arrows) in the maternal blood space (MBS); arrowheads point to IE. FC: fetal capillary. Scale bar: 100 µm (**A,B**); 50 µm (**D,F**).

Identification of CFP^+^ fetal-derived tissue (Figure 2A) was found using intravital imaging of the placenta in the transversal plane. The movement of the blood cell mass in Rhodamine^+^ areas revealed that maternal blood flow was highly heterogeneous in the labyrinth (Video S1). Time-lapse imaging showed maternal blood spaces with high flow rates but also various regions with low flow rates or no perfusion as evidenced by an uneven Dextran-Rhodamine distribution, suggesting that infected placenta display local impairments in microcirculation and tissue perfusion. These heterogeneous blood flow patterns allowed identification of areas with different flow within one microscope field (Figures 2B and 2C).

**Figure 3:**
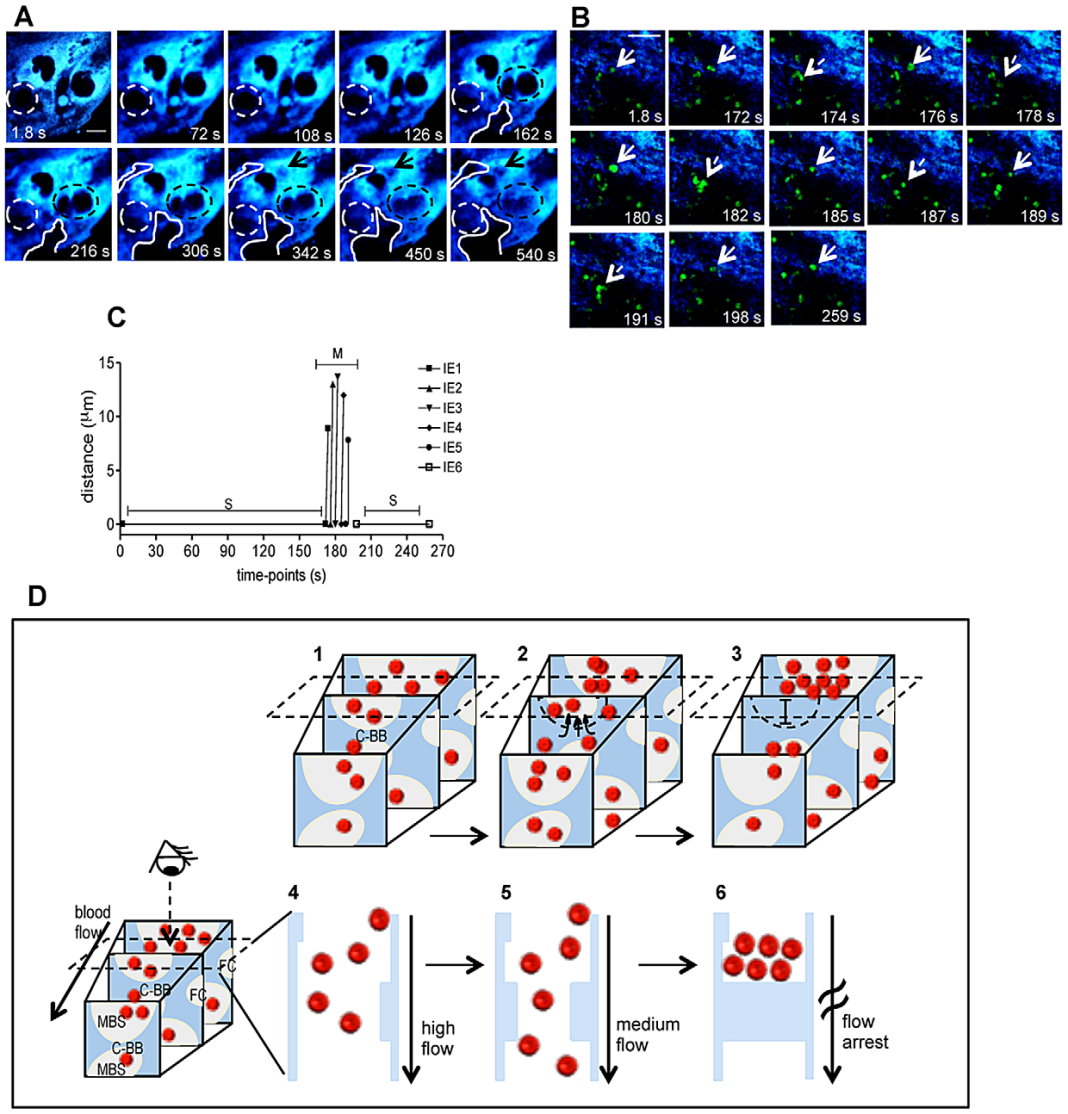
Trophoblast topology dynamics transiently remodels maternal blood spaces. (**A**) Intravital sequential images showing gradual occlusion (dotted circle) and opening (solid line and arrow) of blood space areas (black regions); in blue placental tissue (see Video S3); (**B**) Sequential images of a 4.3 min intravital acquisition showing intermittent blood flow as represented by IE stop-go movement: full line arrows point to IE before moving while the dashed arrow indicates movement (see Video S4). (**C**) Tracking of individual IE (indicated in b) show distance travelled by infected cells in the movement phase (M) alternating with stationary phases (S). (**D**) Diagram illustrating blood flow disturbance by transient trophoblast topological changes that occlude maternal blood space (MBS) and interrupt blood flow (1 to 3); dashed rectangle indicates the imaging plane incidence and shows only events captured on that plane by the 2-photon microscopy (4–6); the third dimension is inferred by compiling sequential images. Possible involvement of “Coan-Burton” bridges is illustrated (C-BB). Red dots: erythrocytes; dashed semi-circle delimits MBS undergoing occlusion; FC: fetal capillary; in blue, placental tissue. Scale bars 20 µm. Illustration of placental tissue fraction (**D**) was adapted from scanning electron micrograph from Coan *et al.* [18].

At higher magnification, blood flow in the labyrinth showed unexpected dynamics. Within the intravital observation period (300 s), blood flow appeared to be interrupted by transient occlusion of the maternal blood space lumen. Figure 2D shows sequential images of a continuous 6 min image acquisition (Video S2) where trophoblast (in blue) and maternal blood space (black areas with GFP^+^ IE) are readily identified. These images demonstrate that blood flow was interrupted after 2 minutes of acquisition as the blood space was occluded by trophoblast conformational changes. Strong co-localization of the 2 images at observation time-points 72 and 360 s indicates that the modifications occurred in the same focal plane and are highly dynamic (Pearson's r = 0.84; Spearman's r = 0.86 - “Pearson-Spearman correlation colocalization” (PSC) test) (Figure 2E). Topological trophoblast changes could involve the cytotrophoblastic bridges (“Coan-Burton bridges”) that cross the maternal blood spaces (Figure 2F), which are abundant during the last third of pregnancy, as recently described by Coan *et al.* [20]. Further, remodeling of maternal blood spaces appears to be transient and seems to operate by reversible constriction. This is illustrated by comparison of sequential images in which trophoblast undergo conformational changes leading to either the “sealing-off” of lumen or “opening up” of other blood spaces (Figure 3A; Video S3). Intermittent passage of individual infected cells within maternal blood spaces suggests that trophoblast can also operate by temporarily “constricting” and “relaxing” the lumen of maternal blood area, thereby controlling blood flow (Figures 3B and 3C; Video S4). We propose that trophoblast topological changes act as highly dynamic and reversible septa that transiently occlude the maternal blood spaces and deflect blood flow (Figure 3C), presumably promoting fetal-maternal exchanges and contributing to heterogeneous maternal blood flow rate patterning across the labyrinth.

### Blood flow heterogeneity in non-infected placenta

**Figure 4:**
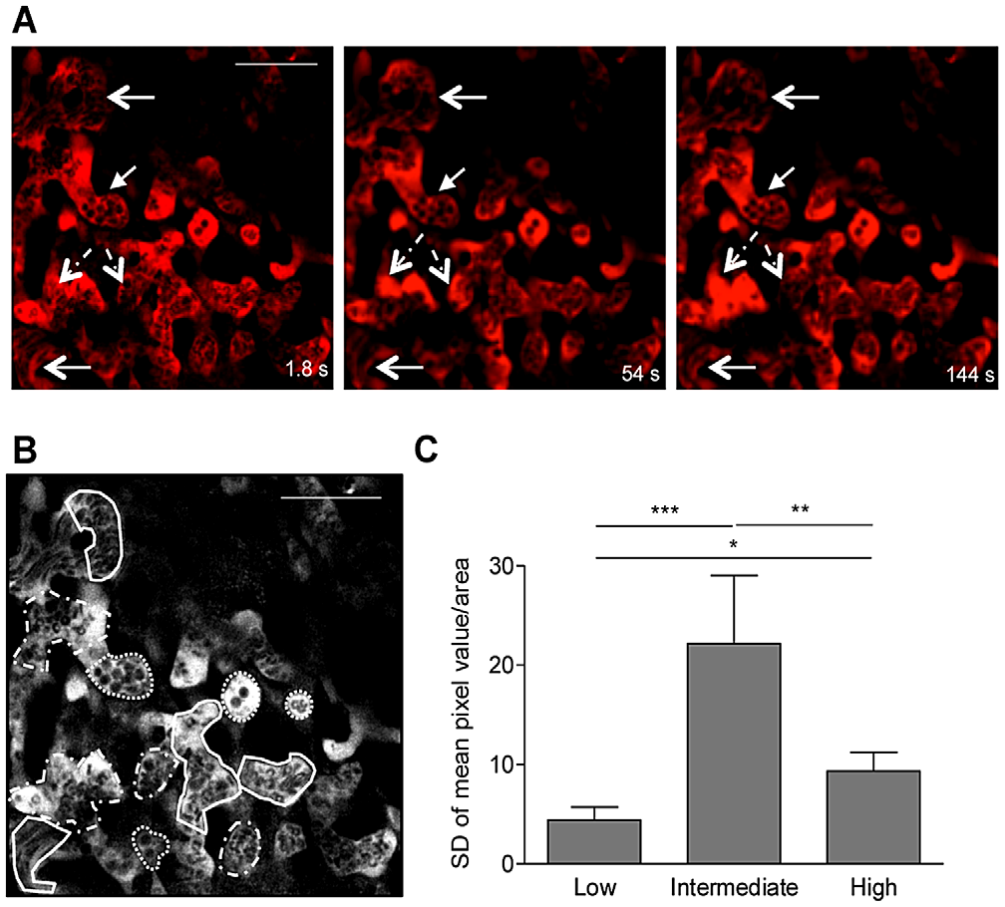
Blood flow heterogeneity in non-infected placenta. Placental imaging was performed on G18 in non-infected pregnant BALB/c mouse as described in Figure 2. (A) Three different time points of a 5 min intravital acquistion (600×) showing regions of low (full line arrows), intermediate (dashed line arrows) or high blood flow (open arrowhead); blood fluid was labeled with Dextran-Rhodamine (red) and blood cells are in black (see Video S5); (B) Areas of high (full line), intermediate (dashed line) and low flow (dotted line) were delimited within the maternal blood space for quantification of mean pixel values in each frame using Fiji imageJ and data was plotted in (C). *p<0.05; **p<0.01; ***p<0.0001 (one-way ANOVA with Tukey's multiple comparison test). Scale bar: 50 µm.

Maternal blood flow patterns were evaluated in the labyrinth of non-infected placentas at late stage gestation (G18). Intravital imaging with a 5 min time-period at 600× magnification showed that Dextran-Rhodamine labeled maternal blood exhibited different flow rates (Figure 4A; Video S5). To quantify the relative blood flow in selected regions we used the Dextran-Rhodamine signal as a reference and analyzed the variance of mean pixel value (MPV) at all time-points in visually defined areas of low, intermediate and high flow (Figure 4B). For this analysis we applied a custom imaging stabilization algorithm that minimizes background movements of the living tissue in the final resulting image sequences (see materials and methods). As expected, areas of low blood flow showed decreased MPV standard deviation (SD) compared to high and intermediate flow areas due to a more constant and lower flow dynamics (Figure 4C). Conversely, the intermediate flow areas had the highest MPV SD, which results from a more heterogeneous flow pattern across the acquisition time-period (very often related to “stop and go” flow). On the other hand, in areas of high flow the MPV SD was increased compared to low flow and decreased compared to areas of intermediate flow as a result of a more constant but highly dynamic flow pattern. These observations confirm that maternal blood flow heterogeneity in the labyrinth is a physiological characteristic of the microcirculation in the mouse placenta.

### IE preferentially accumulate in areas of low blood flow

**Figure 5:**
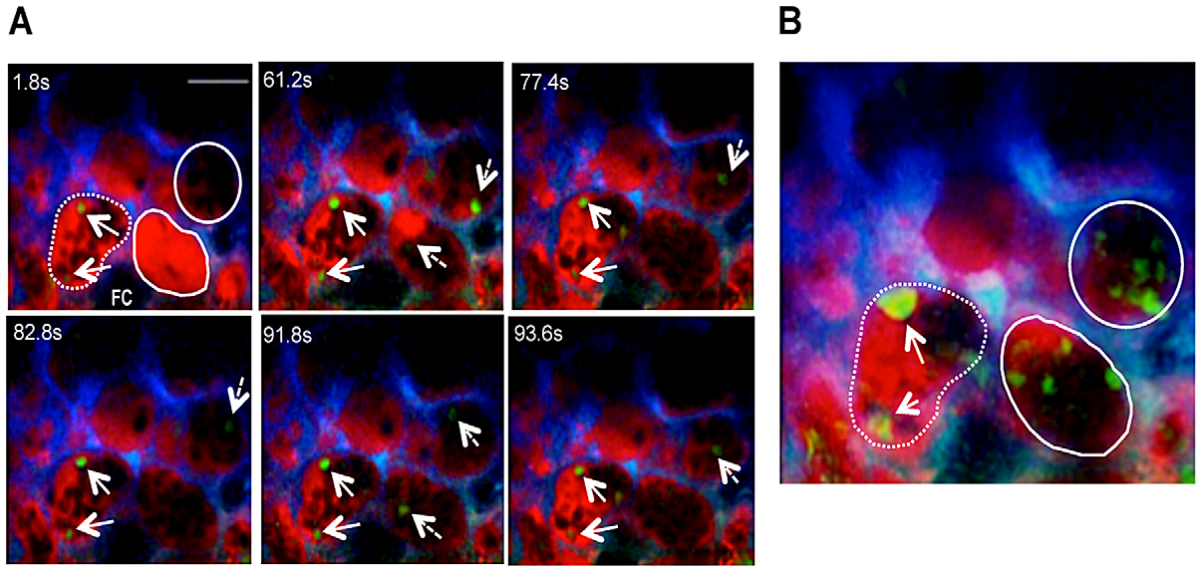
Blood flow determines movement of infected erythrocytes in the placental labyrinth. Placental imaging was performed on G18 in infected pregnant BALB/c mouse. (A) Sequential time-points of a 1.6 min intravital acquisition (Video S6) showing IE that are stationary (full line arrows) in areas of low blood flow (dashed line) or moving (dashed arrows) in high flow areas (full line); (B) Maximum intensity projection along the z axis of sequential frames collected during 37.8 s (55.8 s to 93.6 s; illustrated in A); arrows point to stationary IE in low flow area (as depicted in A); in high blood flow areas (full line) various IE represents parasite movement across time. Z-project was performed using Fiji imageJ. Red: Dextran-Rhodamine labeled blood fluid; green: IE; blue: CFP+ trophoblast; FC: fetal capillary. Scale bar: 30 µm.

We evaluated whether IE movement in infected placentas paralleled blood flow heterogeneity as ascertained by labeling maternal blood with Dextran-Rhodamine (Figure 5A and Video S6). Sequential time-points revealed that IE movement was dictated by blood flow rate. In areas of high blood flow no stationary IE were observed whereas in low flow regions IE remained stationary. Moreover, compilation of images across the z axis confirmed the conserved positioning of IE in low flow areas (as detected by single signals with high intensity) contrasting with the multiple signals of lesser intensity observed in regions of high blood flow (Figure 5B).

**Figure 6:**
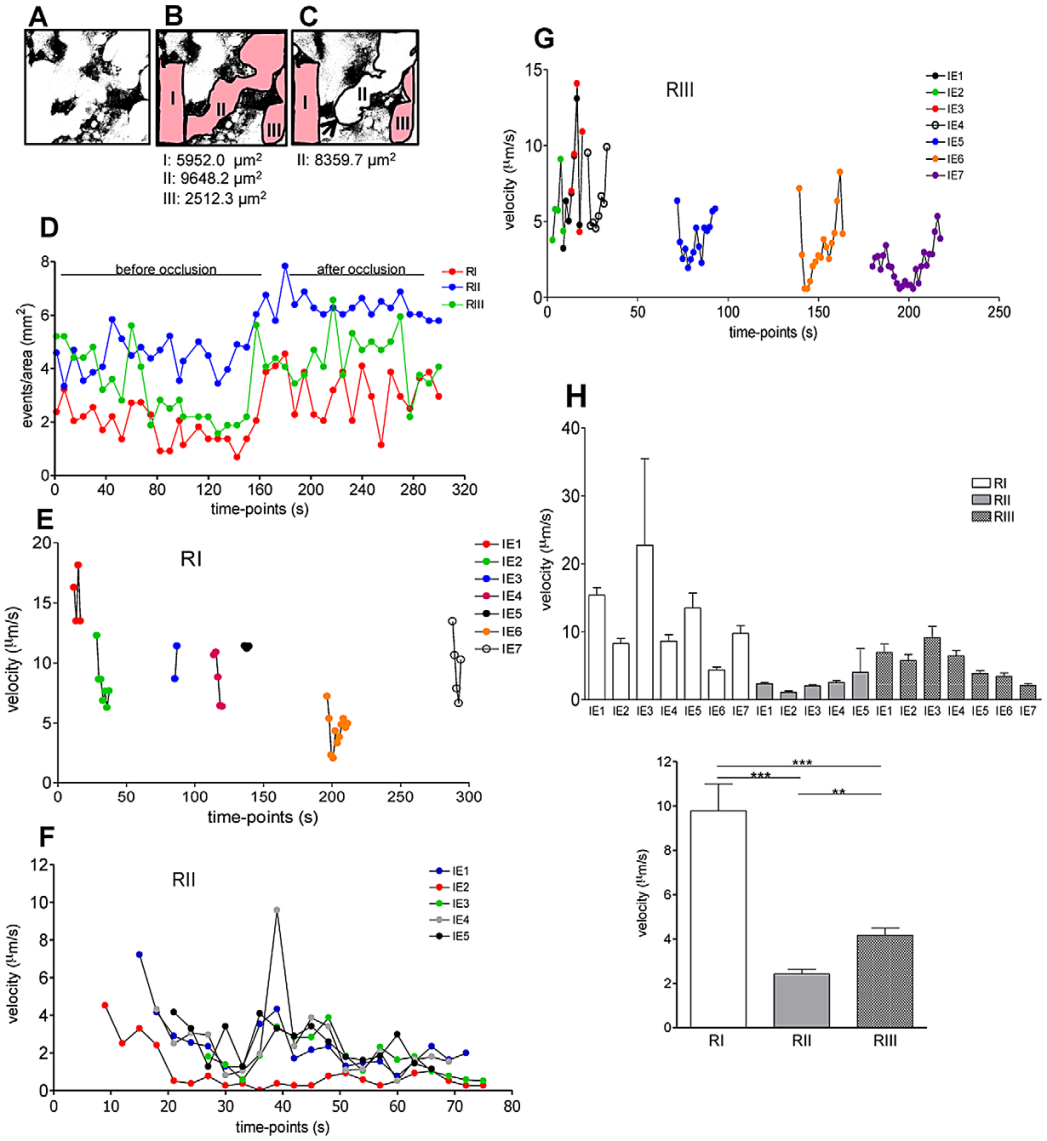
IE accumulation in the labyrinth is favored in regions of low blood flow. Placental imaging was performed on G18 in pregnant BALB/c mouse after infection with *P. berghei-*ANKA at G13. (**A**) Binary images with maternal blood spaces in white and trophoblast in black; maternal blood regions were delimited (red) before (**B**) and after (**C**) occlusion by a “Coan-Burton bridge” (arrow) (see Video S2) interrupting flow (white area). (**D**) Number of IE/region at each 5 time-points was recorded over a 300 s period. Tracking of individual IE was performed during the entire acquisition period in RI (**E**) and RIII (**G**) and during the first 75 s in RII (**F**). (**H**) Average velocity of individual (top) or grouped (bottom) IE in the indicated regions (one-way ANOVA with Tukey's post test; **p<0.01; ***p<0.001).

As our observations suggested that blood flow rate impacted on the speed of IE travelling in the labyrinth we evaluated whether the IE burden within blood spaces differed according to flow rates. From Video S2, we visually identified three different blood flow rates within the same microscopic field (Figures 6A–C) and counted the number of IE per area during an observation period of 6 min (Figure 6D). Time-lapsed analysis showed that the number of IE (events/mm^2^) was consistently higher in regions of lower blood flow rate (RII). This was particularly apparent after blood space occlusion (Figure 2D; Video S2), indicating a preferential IE accumulation in this region compared to RI and RIII (Figure 6D). Individual IE tracking was performed in RI and RIII during the entire observation period whereas in RII IE were followed during the first 75 s of acquisition – the period before blood space occlusion (Figures 6E–G). In RI IE traveled at relatively high speed and could be observed during a period of 10–15 s (Figure 6E) whereas in an intermediate flow region (RIII) IE were observed during 30–40 s (Figure 6G). In contrast, in the RII region IE were present for a longer period (70 s) and velocity was significantly lower compared to RI and RIII (Figure 6F and H). The average velocity of parasitized cells (Figure 6H) confirmed that blood flow rates were sharply distinct amongst these regions. Taken together these observations indicate that transient blood flow alterations in the labyrinth determine the accumulation of IE in areas of low but not high blood flow, possibly favoring IE interactions with the trophoblast layers. These data also suggest that the parasite takes advantage of the heterogeneous placental blood flow pattern to accumulate in the mouse placenta.

### IE-trophoblast interaction dynamics

**Figure 7:**
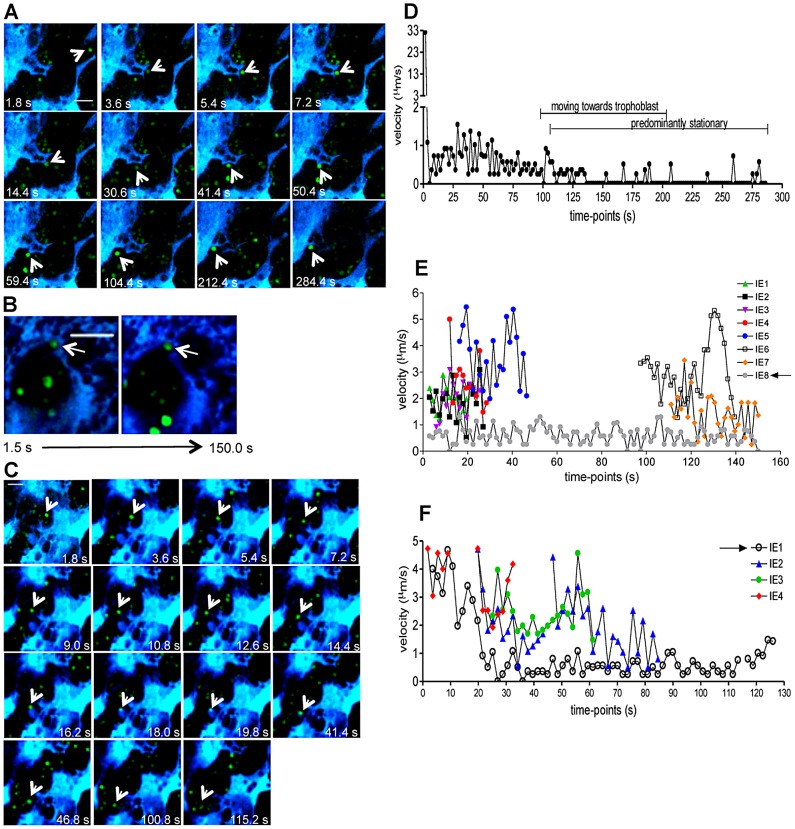
Stationary behavior of parasitized erythrocytes in the placenta. Intravital imaging was performed on G18, five days after infection with *P. berghei*-ANKA GFP+ IE. (**A**) Sequential images of an infected cell (arrow) that moves towards the trophoblast and progressively acquire stationary behavior (2 last images) (see Video S7); (**B**) Two images within 150 seconds interval showing stationary IE (arrow) in a “niche” while other IE travel according to blood flow (see Video S8); (**C**) Images show infected cell (arrow) in transient contact with fetal-derived tissue structure for approx. 80 s (see Video S9); (**D,E,F**) Velocity plots of IE depicted in **A,B** and **C**, respectively; arrows in the legends refer to IE indicated in the respective images (arrow). Infected cells were tracked in images from the videos to ascertain individual cell velocity. Blue: placental tissue; green: IE Scale bar: 20 µm.

Cytoadherence and sequestration of *P. falciparum*-IE in the human placenta via interaction with C4S on trophoblast surface was initially proposed by Fried and Duffy [13] as a key event in placental malaria pathogenesis. *In vitro* studies in PM mouse models also showed binding of *P. berghei* parasitized cells to non-infected placental tissue [14], [19]. By examining intravital images of infected placentas, we observed distinct IE-trophoblast interaction patterns in low blood flow areas. Stable contacts were observed when IE freely travelled in the maternal blood space and encountered the trophoblast. In a typical case the IE experiences a sharply decreased velocity, possibly caused by a rolling contact with the trophoblast membrane, and subsequently attains a steady position. This position was maintained for at least 100 s, until the end of the observation period (Figures 7A and 7D; Video S7). In other cases IE remained stationary, seemingly nested in the trophoblast, suggesting intimate contact with the tissue while other infected cells travelled freely in the blood with velocities varying between 1 and 6 µm/s (Figure 7B and 7E; Video S8). These observations indicate that IE-trophoblast contacts resist the forces of the surrounding blood flow, suggesting strong attachment mechanisms that maintain the IE immobilized on trophoblast membrane. We also observed transient interactions when IE engaged in short-term contact with the trophoblast (aprox. 80 s) followed by disengagement (Figure 7C and 7F; Video S9), possibly representing a failed IE adhesion event.

In many instances, images showed interactions occurring in cytotrophoblast discontinuities (“holes” described by Coan *et al*. [18]), suggesting direct contacts of IE with the underlying trophoblast layers (Figure 7B). By using the same imaging acquisition set-up, we observed that IE do not accumulate on, or interact with, blood vessel walls of the popliteal lymph node in an infected non-pregnant female (Figure S2 and Video S10), thereby validating that IE interact with the placental tissue in distinct manners as compare to peripheral vessels. This data demonstrates that the IE adhere to placental tissue and establish stable interactions that immobilize IE on the trophoblast layers *in vivo* and possibly elicit interhaemal membrane responses.

### Fate of stationary IE

**Figure 8:**
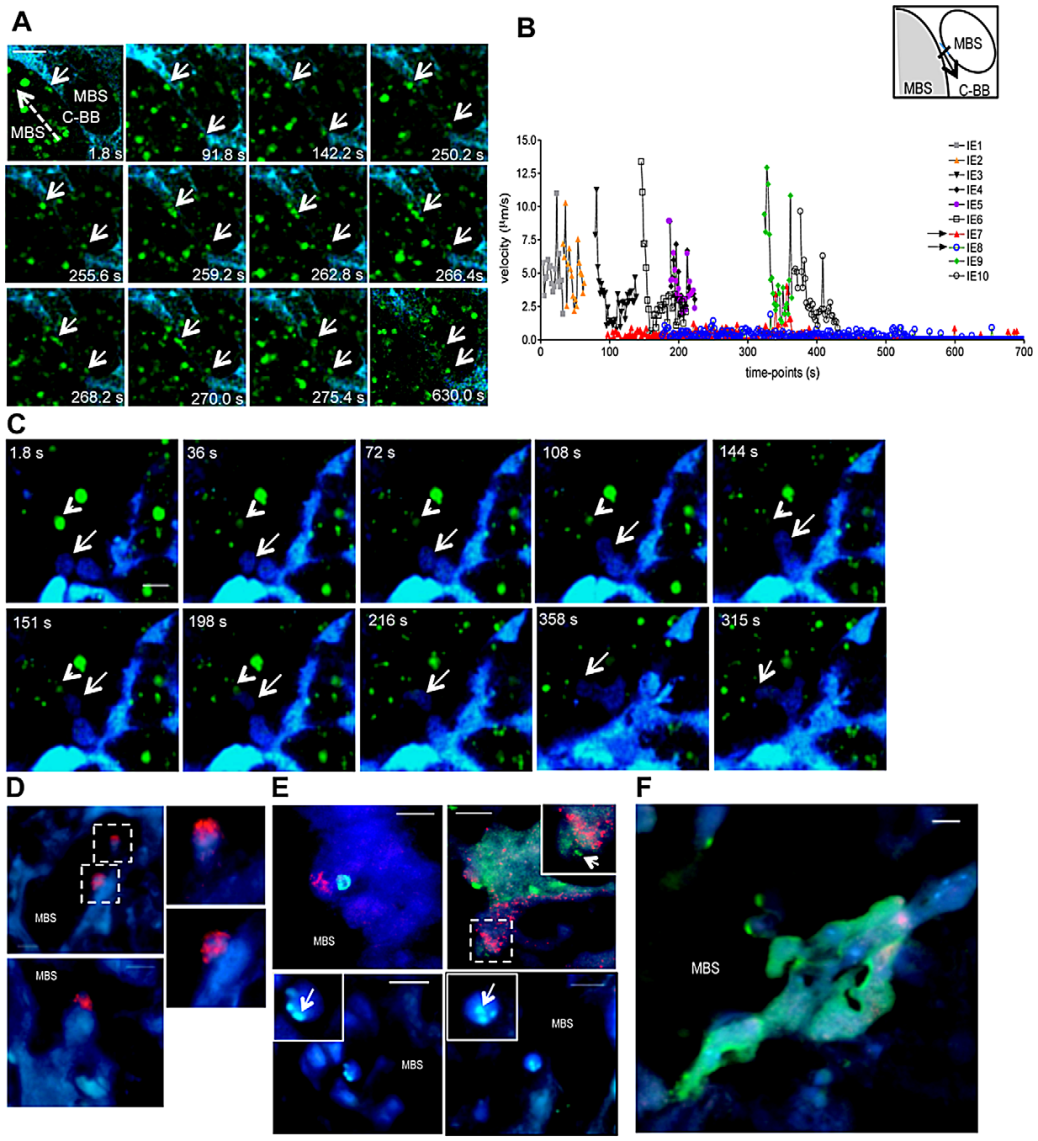
Fate of stationary parasitized erythrocytes in the placenta includes migration across blood spaces and phagocytosis. (**A,B,C**) Intravital imaging was performed on G18, five days after infection with *P. berghei*-ANKA GFP^+^ IE. (**A**) Sequential images show stationary IE (arrows) that traverse the “Coan-Burton bridge” (C-BB) that divide maternal blood space (MBS) remaining stationary in that site (see Video S11); (**B**) Velocity plot of IE depicted in **A**; arrows in the legends refer to IE indicated in the image. Dashed arrow indicates direction of flow; velocity of IE was calculated in the area highlighted in the diagram. (**C**) Sequential images show fetal-derived phagocyte (arrow) migration towards an IE (arrowhead) and engulfment of the infected cell (see Video S12). (**D,E,F**) Immunohistochemistry of *P. berghei*-ANKA GFP^+^ infected placental sections (G18) stained with biotinilated anti-Mac-1 antibody developed with streptavidin-HRP Cy3 conjugate. (**D**) Mac-1^+^ fetal–derived cells oriented towards MBS; images in dashed squares are magnified (upper and lower right). (**E**) Infected erythrocytes within Mac-1^+^ fetal-derived cells (upper left and upper right inset) and Mac-1^−^ fetal cells (upper right, lower right and lower left), magnified in insets (arrows point to GFP^+^ parasite-derived material). (**F**) Parasite-derived material within syncytiotrophoblast layer. Blue: CFP^+^ fetal-derived placental tissue; green: IE, red: Mac-1. Scale bar: 20 µm (A and C) and 10 µm (D, E F).

It is assumed that sequestered IE burst and release infective merozoites, however the fate of stationary IE after attachment to the trophoblast has not yet been addressed. We repeatedly observed that immobilized IE are subjected to further interactions with placental tissue. Image sequences showed that IE were immobilized on the trophoblast for approximately 250 s, abruptly traversed into a neighbor maternal space, and subsequently remained stationary (Figures 8A, 8B and Video S11). This behavior was also observed in Video S6 (see arrowheads). Furthermore, intravital imaging of highly infected placenta provided evidence that stationary IE are targeted by fetal phagocytic cells. Sequential images showed that CFP^+^ cells actively migrate and target IE that are then engulfed and presumably undergo intra-phagocytic destruction (Figure 8C and Video S12) over a time period of approximately 5 minutes.

Furthermore, analysis of infected placenta sections showed that fetal-derived macrophages (Mac-1**^+^** cells) protrude into the maternal blood space (Figure 8D). Both fetal-derived Mac-1^+^ and Mac-1^−^ cells showed engulfed IE and contained parasite-derived material (Figures 8E and F). Microscopic examination also suggested that the majority of cells containing parasite material did not express the myeloid Mac-1 marker. These observations suggest that fetal-derived cells in the labyrinth are actively involved in IE uptake (Figures 8E and F), a finding that is consistent with previous reports indicating that murine placental macrophages and trophoblast lineage cells have phagocytic capacity and can ingest IE [21], [22].

This study provides first ever evidence in the living animal that trophoblast conformational changes modulate blood flow in the mouse placental labyrinth promoting accumulation of infected cells and establishment of intimate IE-throphoblast contacts that lead to IE sequestration. These findings support the notion that *P. berghei*-IE adhere *in vivo* to the trophoblast and highlight the fact that fetal-derived placental cells play a role in the response to placental malaria.

## Discussion

Here we provide an intravital description of the *Plasmodium*-infected placenta in the mouse. Our findings reveal novel placental microcirculatory attributes that favor PM pathology and reinforce the current paradigm that IE establish intimate interactions with trophoblasts and elicit fetal-derived cellular responses in the placenta. Specifically, this study demonstrates that unique characteristics of placental microcirculation driven by trophoblast conformational changes favor intra-placental IE accumulation. Furthermore, we show that fetal-derived cells in the placenta are involved in phagocytosis of sequestered IE *in vivo*.

Two-photon microscopy was used to unveil the microcirculatory dynamics in the infected placental labyrinth. The technical procedure for *in vivo* imaging requires anesthesia and surgical exposure of the placenta unit that may influence the organ hemodynamics. Nevertheless our observations are compelling in revealing that placental microcirculation is, at least partially, governed by the fetal-derived trophoblast conformational changes. The trophoblast topology imposes heterogeneous blood flow pattern across the placenta, which is evident in absence of infection. We visualized areas of transient low-level flow or stasis providing intravital evidence that blood flow rate in the placental microcirculation is not controlled by the maternal arterial blood pressure. We propose that transient constrictions of maternal blood spaces generated by reversible trophoblast conformational changes are at the basis of an exquisite mechanism to control blood flow warranting prolonged contact of maternal blood with the interhaemal membrane. It did not escape our attention that some dynamic conformational changes may involve the “Coan-Burton bridges” as these structures were observed and described in an ultra-structural study as cytotrophoblastic prolongations connecting separate sides of maternal blood space lumen [20]. Such bridges could originate transient blood space obliteration and control maternal blood flow as evidenced by our intravital observations and illustrated in Figure 3C. Nevertheless, without a detailed 3D description of the labyrinth architecture at microscopic scale it is difficult to envisage how this flow-control mechanism could be coordinated at placental organ level. Also, a full three-dimensional reconstruction of the placenta would provide further information about the impact of *Plasmodium* infection on microcirculation, correlating the alterations in placental haemodynamics with pathological findings. It is plausible that such hemodynamic alterations would lead to increased uterine and umbilical artery vascular resistance as reported in pregnant women with *P. falciparum* infection [23], [24].

Rhodamine negative maternal blood areas that contained stationary IE were frequently observed, indicating impaired perfusion. The uneven pattern of Rhodamine distribution in these maternal blood spaces is compatible with blood flow interruption (or low perfusion) by fibrin deposition leading to clot formation and subsequent thrombosis. Significant fibrin deposits in the intervillous space have been documented in placental malaria [25]–[27]. In *P. falciparum-*infected placenta fibrin clots narrowed and plugged intervillous spaces [27]. Fibrin thrombi formation and hemorrhage were also observed in placentas of aborted mice that were infected at G0 with *P. chabaudi* [28]. A recent study showed that intrauterine growth restriction was associated with compromised maternal circulation displaying slow intervillous blood flow, intermittent stops in perfusion as well as unperfused regions [29]. In our experimental system we frequently observe fibrin deposition in histological sections (Figure S3) that raises the possibility that micro-thrombotic events can lead to low perfusion and contribute to PM pathology.

Our observations demonstrate that low blood flow favors accumulation of IE in maternal blood spaces and promotes the establishment of stable contacts with the trophoblast. Apart from the CS4 enriched environment, we propose that the presence of low and intermediate flow regions in the placental labyrinth favors IE sequestration. Nevertheless, we noted that the IE encounters with trophoblast structures did not always lead to stable contact since transient IE-trophoblast interactions were also observed. As opposed to *in vitro* binding assays, the intravital images suggest that IE adhesion to the trophoblast is not limited to passive receptor-ligand interaction. The trophoblast appears to actively react to the presence of IE at the margins of maternal blood spaces, as is the case of sequestered IE that were carried across neighboring maternal blood regions (Figure 8A and Video S11).

The observations that stationary IE are targeted by fetal macrophages and trophoblast provide evidence for an active reaction to the presence of IE. Our study is in line with findings showing that fetal placenta cells such as the trophoblast [21], [28], [30]–[33] and placental macrophages [22] respond to parasite components. The exact mechanisms linking fetal-derived placental cellular response against the parasite to the pro-inflammatory environment and angiogenic impairments observed in infected placenta [34]–[36] are yet to be determined.

Our findings lead us to propose that placental hemodynamics as well as trophoblast responses to sequestered IE contribute to PM pathogenesis in the mouse. Despite the marked differences in microanatomy of the mouse and human placenta it cannot be excluded that impairments in microcirculation and pro-inflammatory responses from fetal-derived placental tissue also play a role in human *Plasmodium* placental infection. Nevertheless, it should be noted that the mouse model here studied represents an aggressive and acute form of placental infection that is observed in only a fraction of pregnant women with malaria [37].

## Materials and Methods

### Ethics statement

All procedures involving laboratory mice were in accordance with national (Portaria 1005/92) and European regulations (European Directive 86/609/CEE) on animal experimentation and welfare and were approved by the Instituto Gulbenkian de Ciência Ethics committee and the Direção-Geral de Veterinária is the Official National Entity that regulates the use of laboratory animals in Portugal.

### Animals and parasites

Eight to twelve week-old BALB/c female and B6-Tg(CAG-ECFP) (B6-Cyan) male mice were obtained from the Instituto Gulbenkian de Ciência animal facility. Mice were bred and maintained under specific-pathogen free (SPF) conditions. All infection experiments made use of *P. berghei* ANKA constitutively expressing green fluorescent protein under the eef-1 promoter (ANKA-GFP) [38], [39]. Infection inocula consisted of infected erythrocyte preparations (IE) obtained after one *in vivo* passage of a frozen parasite stock that was injected intra-peritoneally in BALB/c mice and collected when parasitemia reached approximately 10%.

### Pregnancy monitoring and infection

BALB/c females were mated to B6-Cyan males to obtain placentas where fetal components expressed cyan fluorescent protein (CFP). The day that the pair was separated was considered gestational day 1 (G1). Pregnancy was monitored every other day by weighing the females. Body weight gain of 3 to 4 g at G10 to G13 indicated successful fertilization. Pregnant mice were intravenously (i.v.) infected on G13 with 10^6^ *P. berghei* ANKA-GFP IE [14]. Intravital imaging was performed on G18. Intravital images of lymph-node blood vessels from non-pregnant females were acquired 7 days after infection with 10^6^ ANKA-GFP^+^ IE.

### Placenta preparations for histology

Placentas from infected and non-infected pregnant mice, sacrificed on G18, were fixed in 10% formalin and embedded in paraffin. Non-consecutive 5 µm sections were stained with hematoxylin-eosin (HE) and examined under light microscope (Leica DM LB2, Leica Microsystems).

### Immunohistochemistry

Placentas from *P. berghei*-GFP^+^ infected BALB/c females that were mated to B6.Cyan males were fixed overnight in 4% formalin/6% sucrose, embedded in Tissue Tek OCT compound (Sakura), snap frozen in liquid nitrogen and cut in 7 µm-thick slices using a Leica 3050S cryostat (Leica Microsystems, Germany). Sections were rehydrated for 10 min in PBS 1X, stained with rat anti-mouse Mac-1(M1/70) biotinilated antibody and developed with streptavidin-HRP Cy3 conjugate. All incubations were performed at room temperature with PBS1x/10% FCS/0.1% azide/5% mouse serum. Slides were mounted in 2.5% 1,4-Diazabicyclo (2,2,2) octane (pH 8.6) in 90% glycerol in PBS and images were acquired in a DMRA2 Leica microscope (Leica Microsystems) using 63X and 100X objectives.

### Surgical procedures and microscopy staging

Mice were anaesthetized with 150 mg ketamine and 12 mg xylazine per kg body weight and kept on a warm pad at 37°C. For placental imaging, an incision on the lower abdomen was performed and one feto-placental unit of the uterus exposed. Uterine membrane was gently incised and fetus and placenta liberated so that placenta could be exposed in its entirety whilst still attached to the uterus by the decidua, so not compromising tissue irrigation. The fetus was covered with gauze immersed in PBS to avoid drying. Mouse was restrained in a bespoke apparatus with a sliding lid and the placenta was immobilized with a clip to display the fetal side upwards. The placenta was stabilized for observation by covering with a metal platform with an orifice in the middle which holds a cover slip. Tissue hydration was assured by surrounding the tissue with 2% low melting agarose and temperature was monitored with a sensor placed in contact with the tissue. This procedure is described in more detail by Zenclussen *et al* [40]. Preparation of popliteal lymph nodes was performed as previously described [41] Briefly, hind legs were shaved, the animal restrained on a warm pad at 37°C and an incision on the back of the hind leg near the “K” shaped artery was performed. The lymph node was exposed and immobilized using a metal strap with a small orifice. For blood flow detection, mice were injected i.v. with 1 mg of Rhodamine B isothicyanate (Dextran-Rhodamine) (Sigma-Aldrich) diluted in PBS, immediately before imaging.

### Two-photon microscopy

A Praire Ultima two-photon microscope on an Olympus BX-51 base with x-y translation stage equipped with two sets of conventional galvanometer-based scanners, fitted with a 2P Coherent Chameleon Laser tuned to 900–910 nm was used throughout. Rhodamine signal was separated from both GFP and CFP fluorescence emission using a dichroic mirror of 565 nm. The GFP/CFP fluorescent protein signals were split by a 495 nm dichroic mirror. Filters used were 500–550 (GFP), 435–485 (CFP) and 570–620 nm (Rhodamine). Time-lapse imaging of a single focal plan was performed. Data were acquired using PraireView software and 2D T-series imaging was performed in 512×512 pixel size frame at a rate of 1.8 s/frame. Objectives used were 20X (1.0 NA 2 mm working distance) and 60X (0.90 NA 2 mm working distance). Images were processed and data analyzed using Fiji/ImageJ 1.46a software (http://pacific.mpi-cbg.de). Cell velocity and distance travelled by infected cells were calculated using the Fiji/ImageJ Manual Tracking plug-in.

### Image stabilization

Due to endogenous motion, such as that caused by intestinal peristaltic movement, we performed a software-based post-processing step to stabilize the intravital image sequences over time in order to better quantify the blood flow hemodynamics inside the labyrinth. For this, we developed a custom software algorithm that is conceptually simple and efficient and that has the capability to stabilize a full 3D two-photon microscopy image sequence. In particular, our software (to be published elsewhere) performs a cross-correlation based image registration between two consecutive z-image stacks and provides the optimal displacement (x,y,z), such that the global pixel overlap between these two image stacks is maximum. Simply stated, the image drift in all dimensions (x, y, and z planes) is minimized in order to achieve a stable and better movie quality. In this study, only 2D image acquisitions were performed. Nonetheless, we were able to use our software to stabilize these image sequences with the same principles described above.

### Image processing

In non-infected placentas, areas were selected by visual inspection based on apparent flow rate. A constant region of interest (ROI) was defined and the mean pixel value (MPV) was calculated for each frame in the image sequence. Also, the standard deviation (SD) of the MPV/frame was calculated. In infected placentas, an output image was generated from the merging of all images along the z axis containing the maximum pixel values over all images in the stack. This image analysis procedure was performed using Fiji/ImajeJ 1.46a software.

### Statistical analysis

Data were presented as mean values +/− SEM. Unpaired *t* test or ANOVA with Tukey's or Dunnet's post-test were performed using the GraphPad Prism 4.0 software. Data were considered significant for *p*<0.05. Pearson and Spearman correlation coefficients were calculated using a specific plug-in (http://www.cpib.ac.uk/~afrench/coloc) [42] of Fiji/ImageJ 1.46a image processing software.

## Supporting Information

Figure S1 Diagram of the experimental procedure. BALB/c females were mated to B6-Cyan for 48 hours. Pregnant mice were infected with *P. berghei-*ANKA GFP^+^ IE on G13. On day 5 post-infection, mouse was anesthetized, placenta was exposed and maternal blood fluid was labeled (when applicable) with an i.v. injection of Dextran-Rhodamine immediately before imaging. Images were acquired in a single focal plan at a rate of 1.8 s/frame using a *Praire Ultima* two-photon microscope.
(DOCX)

Figure S2 Parasitized erythrocytes in the periphery. Non-pregnant BALB/c female was infected with *P. berghei-*ANKA and imaged 1 week after infection. Sequential images of blood circulation inside the popliteal lymph-node show IE (indicated by arrows) travelling at speed over 20 µm/s (velocity was calculated by Fiji imageJ manual tracking plug-in; data not shown) (see Video S10). Red: blood-labeled Dextran-Rhodamine; green; IE. Scale bar: 50 µm.
(DOCX)

Figure S3 Deposition of amorfous eosinophilic fibrinoid material in the lumina of maternal blood space (MBS) (**A**), often associated with necrotic cell debris (**B**) (arrows). Thrombus formation was also registered (**C**). H&E sagittal section of *P.berghei*-infected placentas at G18. FC: fetal capillary; IM: interheamal membrane.
(DOCX)

Video S1 Distinct blood flow rates in the infected placenta. Intravital acquisition of *P. berghei*-infected mouse at G18 showing fetal-derived CFP^+^ placental tissue (blue), GFP^+^ IE (green) and Dextran-Rhodamine^+^ labelled blood fluid (red). Images were acquired during 3 min with a 20X objective. Scale bar: 100 µm; images are at 5 frames/s.
(AVI)

Video S2 Maternal blood flow is conditioned by trophoblast. Magnified area of the infected placenta (600X) at G18 shows blood flow interruption by emergence of a trophoblast bridge (arrow). Images were acquired during 6 min. Placental tissue (blue), GFP^+^IE (green); blood spaces (black). Scale bar: 50 µm; images are at 5 frames/s.
(AVI)

Video S3 Intermittent blood flow in maternal space of infected placenta. Sequential images (4.3 min) show GFP^+^IE alternating between stationary and progressive movement. Placental tissue (blue), GFP^+^IE (green); maternal blood spaces (black). Arrow indicates region of the referred events. Scale bar: 50 µm; images are at 5 frames/s.
(AVI)

Video S4 Trophoblast can occlude blood space lumen. Intravital imaging (9 min) of infected placenta show gradual occlusion (white arrow) and opening of blood space areas (black regions; green arrow); blue: placental tissue. Scale bar: 20 µm; images are at 10 frames/s.
(AVI)

Video S5 Blood flow pattern of non-infected placenta. Intravital imaging (5 min) of the placental labyrinth at G18 shows heterogeneous blood flow rates. Blood fluid was labeled with Dextran-Rhodamine (red); black regions inside blood spaces are erythrocytes. Scale bar: 30 µm; images are at 5 frames/s.
(AVI)

Video S6 IE movement in high and low blood flow regions. Images were acquired during 1.6 min and show stationary parasites (black arrow) only in low blood flow regions. Arrowhead points to IE that traverses maternal adjacent blood spaces. Placental tissue (blue), GFP^+^IE (green), Dextran-Rhodamine labeled maternal blood (red), FC (fetal capillary). Scale bar: 30 µm; images are at 5 frames/s.
(AVI)

Video S7 *P. berghei*-infected cell shows progressive stationary behavior. Five-minute imaging show parasitized cell moving towards the trophoblast with apparent contact and progressively acquiring stationary behavior (arrow). Placental tissue (blue), GFP^+^IE (green). Scale bar: 20 µm; images are at 10 frames/s.
(AVI)

Video S8 Stationary infected cells in a “niche” (arrows) away from blood flow. Placental tissue (blue), GFP^+^IE (green). Scale bar: 20 µm; images are at 10 frames/s (4.5 min acquisition).
(AVI)

Video S9 Transient contact of parasitized cell with placental tissue. Sequential images show IE caught in a fetal-derived placental cell for approx 80 s followed by a possible escape behavior (arrow). Placental tissue (blue), GFP^+^IE (green). Scale bar: 20 µm; images are at 5 frames/s (2 min acquisition).
(AVI)

Video S10 Parasitized erythrocytes in the popliteal lymph-node blood circulation. Non-pregnant BALB/c female was infected with *P. berghei-*ANKAGFP^+^ and imaged 1 week after infection. Arrows point to infected cells. Green: IE; red: blood fluid. Scale bar: 20 µm; images are at 5 frames/s (1.5 min acquisition).
(AVI)

Video S11 IE migrate across blood spaces. Sequential images (7.2 min) show stationary IE (arrows) that trasverse the “Coan-Burton bridge” (C-BB) and migrate to the adjacent maternal space (MBS) remaining stationary in that site. Placental tissue (blue), GFP^+^IE (green). Scale bar: 20 µm; images are at 5 frames/s.
(AVI)

Video S12 Stationary cells are target of phagocytosis. Sequential images show fetal-derived phagocyte (arrow) migrating towards an IE (arrowhead) and engulfing the infected cell. Blue: placental tissue; green: IE. Scale bar: 20 µm; images are at 10 frames/s (6 min acquisition).
(AVI)

## References

[1] World Health Organization. A Strategic Framework for Malaria Prevention and Control During Pregnancy in the African Region. (2004).

[2] Duffy PE, R.S. Desowitz RS (2001) Pregnancy malaria throughout history: dangerous labors. Malaria in Pregnancy. Deadly Parasite, Susceptible Host. Taylor and Francis 1–25.

[3] Achur RN, Valiyaveettil M, Alkhalil A, Ockenhouse CF, Gowda DC (2000) Characterization of proteoglycans of human placenta and identification of unique chondroitin sulfate proteoglycans of the intervillous spaces that mediate the adherence of Plasmodium falciparum-infected erythrocytes to the placenta. J Biol Chem 275: 40344–40356. 11005814 10.1074/jbc.M006398200

[4] Muthusamy A, Achur RN, Bhavanandan VP, Fouda GG, Taylor DW, et al (2004) Plasmodium falciparum-infected erythrocytes adhere both in the intervillous space and on the villous surface of human placenta by binding to the low-sulfated chondroitin sulfate proteoglycan receptor. Am J Pathol 164: 2013–2025. 15161637 10.1016/S0002-9440(10)63761-3 PMC1615783

[5] Salanti A, Staalsoe T, Lavstsen T, Jensen AT, Sowa MP, et al (2003) Selective upregulation of a single distinctly structured var gene in chondroitin sulphate A-adhering Plasmodium falciparum involved in pregnancy-associated malaria. Mol Microbiol 49: 179–191. 12823820 10.1046/j.1365-2958.2003.03570.x

[6] Duffy MF, Maier AG, Byrne TJ, Marty AJ, Elliott SR, et al (2006) VAR2CSA is the principal ligand for chondroitin sulfate A in two allogeneic isolates of Plasmodium falciparum. Mol Biochem Parasitol 148: 117–124. 16631964 10.1016/j.molbiopara.2006.03.006

[7] Suguitan AL, Leke RG, Fouda G, Zhou A, Thuita L, et al (2003) Changes in the levels of chemokines and cytokines in the placentas of women with Plasmodium falciparum malaria. J Infect Dis 188: 1074–1082. 14513430 10.1086/378500

[8] Abrams ET, Brown H, Chensue SW, Turner GD, Tadesse E, et al (2003) Host response to malaria during pregnancy: placental monocyte recruitment is associated with elevated beta chemokine expression. J Immunol 170: 2759–2764. 12594307 10.4049/jimmunol.170.5.2759

[9] Khusmith S, Druilhe P, Gentilini M (1982) Enhanced Plasmodium falciparum merozoite phagocytosis by monocytes from immune individuals. Infect Immun 35: 874–879. 7040248 10.1128/iai.35.3.874-879.1982 PMC351128

[10] Walter PR, Garin Y, Blot P (1982) Placental pathologic changes in malaria. A histologic and ultrastructural study. Am J Pathol 109: 330–342. 6758604 PMC1916118

[11] Bulmer JN, Rasheed FN, Morrison L, Francis N, Greenwood BM (1993) Placental malaria. II. A semi-quantitative investigation of the pathological features. Histopathology 22: 219–225. 8495955 10.1111/j.1365-2559.1993.tb00111.x

[12] Leopardi O, Naughten W, Salvia L, Colecchia M, Matteelli A, et al (1996) Malaric placentas. A quantitative study and clinico-pathological correlations. Pathol Res Pract 192: 892–8. 8950755 10.1016/S0344-0338(96)80068-9

[13] Fried M, Duffy PE (1996) Adherence of Plasmodium falciparum to chondroitin sulfate A in the human placenta. Science 272: 1502–1504. 8633247 10.1126/science.272.5267.1502

[14] Neres R, Marinho CR, Goncalves LA, Catarino MB, Penha-Goncalves C (2008) Pregnancy outcome and placenta pathology in Plasmodium berghei ANKA infected mice reproduce the pathogenesis of severe malaria in pregnant women. PLoS One 3: e1608. 18270595 10.1371/journal.pone.0001608 PMC2229663

[15] Rodrigues-Duarte L, Vieira de Moraes L, Barboza R, Marinho CR, Franke-Fayard B, et al (2012) Distinct placental malaria pathology caused by different Plasmodium berghei lines that fail to induce cerebral malaria in the C57Bl/6 mouse. Malaria journal 11: 231. 22799533 10.1186/1475-2875-11-231 PMC3485172

[16] Craig AG, Grau GE, Janse C, Kazura JW, Milner D, et al (2012) The Role of Animal Models for Research on Severe Malaria. PLoS Pathog 8: e1002401. 22319438 10.1371/journal.ppat.1002401 PMC3271056

[17] Malassine A, Frendo JL, Evain-Brion D (2003) A comparison of placental development and endocrine functions between the human and mouse model. Hum Reprod Update 9: 531–539. 14714590 10.1093/humupd/dmg043

[18] Watson ED, Cross JC (2005) Development of structures and transport functions in the mouse placenta. Physiology (Bethesda) 20: 180–193. 15888575 10.1152/physiol.00001.2005

[19] Marinho CR, Neres R, Epiphanio S, Goncalves LA, Catarino MB, et al (2009) Recrudescent Plasmodium berghei from pregnant mice displays enhanced binding to the placenta and induces protection in multigravida. PLoS One 4: e5630. 19461965 10.1371/journal.pone.0005630 PMC2680968

[20] Coan PM, Ferguson-Smith AC, Burton GJ (2005) Ultrastructural changes in the interhaemal membrane and junctional zone of the murine chorioallantoic placenta across gestation. J Anat 207: 783–796. 16367805 10.1111/j.1469-7580.2005.00488.x PMC1571584

[21] Poovassery J, Moore JM (2009) Association of malaria-induced murine pregnancy failure with robust peripheral and placental cytokine responses. Infect Immun 77: 4998–5006. 19687196 10.1128/IAI.00617-09 PMC2772554

[22] Pavia CS, Niederbuhl CJ (1991) Immunization and protection against malaria during murine pregnancy. Am J Trop Med Hyg 44: 176–182. 2012261 10.4269/ajtmh.1991.44.176

[23] Arbeille P, Carles G, Bousquet F, Body G, Lansac J (1998) Fetal cerebral and umbilical artery blood flow changes during pregnancy complicated by malaria. J Ultrasound Med 17: 223–229. 9544605 10.7863/jum.1998.17.4.223

[24] Dorman EK, Shulman CE, Kingdom J, Bulmer JN, Mwendwa J, et al (2002) Impaired uteroplacental blood flow in pregnancies complicated by falciparum malaria. Ultrasound Obstet Gynecol 19: 165–170. 11876809 10.1046/j.0960-7692.2001.00545.x

[25] Ismail MR, Ordi J, Menendez C, Ventura PJ, Aponte JJ, et al (2000) Placental pathology in malaria: a histological, immunohistochemical, and quantitative study. Hum Pathol 31: 85–93. 10665918 10.1016/s0046-8177(00)80203-8

[26] Imamura T, Sugiyama T, Cuevas LE, Makunde R, Nakamura S (2002) Expression of tissue factor, the clotting initiator, on macrophages in Plasmodium falciparum-infected placentas. J Infect Dis 186: 436–440. 12134244 10.1086/341507

[27] Muehlenbachs A, Fried M, McGready R, Harrington WE, Mutabingwa TK, et al (2010) A novel histological grading scheme for placental malaria applied in areas of high and low malaria transmission. J Infect Dis 202: 1608–1616. 20929353 10.1086/656723 PMC3006170

[28] Poovassery JS, Sarr D, Smith G, Nagy T, Moore JM (2009) Malaria-induced murine pregnancy failure: distinct roles for IFN-gamma and TNF. J Immunol 183: 5342–5349. 19783682 10.4049/jimmunol.0901669 PMC2772180

[29] Brunelli R, Masselli G, Parasassi T, De Spirito M, Papi M, et al (2010) Intervillous circulation in intra-uterine growth restriction. Correlation to fetal well being. Placenta 31: 1051–1056. 20970852 10.1016/j.placenta.2010.09.004

[30] Lucchi NW, Koopman R, Peterson DS, Moore JM (2006) Plasmodium falciparum-infected red blood cells selected for binding to cultured syncytiotrophoblast bind to chondroitin sulfate A and induce tyrosine phosphorylation in the syncytiotrophoblast. Placenta 27: 384–394. 16009422 10.1016/j.placenta.2005.04.009

[31] Lucchi NW, Peterson DS, Moore JM (2008) Immunologic activation of human syncytiotrophoblast by Plasmodium falciparum. Malar J 7: 42. 18312657 10.1186/1475-2875-7-42 PMC2268702

[32] Lucchi NW, Sarr D, Owino SO, Mwalimu SM, Peterson DS, et al (2011) Natural hemozoin stimulates syncytiotrophoblast to secrete chemokines and recruit peripheral blood mononuclear cells. Placenta 32: 579–585. 21632106 10.1016/j.placenta.2011.05.003 PMC3142316

[33] Chaisavaneeyakorn S, Lucchi N, Abramowsky C, Othoro C, Chaiyaroj SC, et al (2005) Immunohistological characterization of macrophage migration inhibitory factor expression in Plasmodium falciparum-infected placentas. Infect Immun 73: 3287–3293. 15908353 10.1128/IAI.73.6.3287-3293.2005 PMC1111854

[34] Conroy A, Serghides L, Finney C, Owino SO, Kumar S, et al (2009) C5a enhances dysregulated inflammatory and angiogenic responses to malaria in vitro: potential implications for placental malaria. PLoS ONE 4: e4953. 19308263 10.1371/journal.pone.0004953 PMC2655724

[35] Kabyemela ER, Fried M, Kurtis JD, Mutabingwa TK, Duffy PE (2008) Fetal responses during placental malaria modify the risk of low birth weight. Infect Immun 76: 1527–1534. 18212078 10.1128/IAI.00964-07 PMC2292860

[36] Muehlenbachs A, Fried M, Lachowitzer J, Mutabingwa TK, Duffy PE (2008) Natural selection of FLT1 alleles and their association with malaria resistance in utero. Proc Natl Acad Sci USA 105: 14488–14491. 18779584 10.1073/pnas.0803657105 PMC2567167

[37] Hviid L, Marinho CR, Staalsoe T, Penha-Goncalves C (2010) Of mice and women: rodent models of placental malaria. Trends Parasitol 26: 412–419. 20605743 10.1016/j.pt.2010.04.010

[38] Janse CJ, Franke-Fayard B, Mair GR, Ramesar J, Thiel C, et al (2006) High efficiency transfection of Plasmodium berghei facilitates novel selection procedures. Mol Biochem Parasitol 145: 60–70. 16242190 10.1016/j.molbiopara.2005.09.007

[39] Janse CJ, Franke-Fayard B, Waters AP (2006) Selection by flow-sorting of genetically transformed, GFP-expressing blood stages of the rodent malaria parasite, Plasmodium berghei. Nat Protoc 1: 614–623. 17406288 10.1038/nprot.2006.88

[40] Zenclussen AC, Olivieri DN, Dustin ML, Tadokoro CE (2012) In vivo multiphoton microscopy technique to reveal the physiology of the mouse placenta. Am J Reprod Immunol 68: 271–278. 22626451 10.1111/j.1600-0897.2012.01161.x PMC3465783

[41] Fooksman DR, Schwickert TA, Victora GD, Dustin ML, Nussenzweig MC, et al (2010) Development and migration of plasma cells in the mouse lymph node. Immunity 33: 118–127. 20619695 10.1016/j.immuni.2010.06.015 PMC2952879

[42] French AP, Mills S, Swarup R, Bennett MJ, Pridmore TP (2008) Colocalization of fluorescent markers in confocal microscope images of plant cells. Nat Protoc 3: 619–628 Research Support, Non-U.S. Gov’t. 18388944 10.1038/nprot.2008.31

