# Supplementary material for: Intravital Placenta Imaging Reveals Microcirculatory Dynamics Impact on Sequestration and Phagocytosis of Plasmodium-Infected Erythrocytes
Source: PLoS Pathog. 2013 Jan 31;9(1):e1003154. doi: 10.1371/journal.ppat.1003154 (PMC3561179; doi:10.1371/journal.ppat.1003154)
Supplement: Figure S1 — Diagram of the experimental procedure. BALB/c females were mated to B6-Cyan for 48 hours. Pregnant mice were infected with P. berghei-ANKA GFP+ IE on G13. On day 5 post-infection, mouse was anesthetized, placenta was exposed and maternal blood fluid was labeled (when applicable) with an i.v. injection of Dextran-Rhodamine immediately before imaging. Images were acquired in a single focal plan at a rate of 1.8 s/frame using a Praire Ultima two-photon microscope. (DOCX) [file ppat.1003154.s001.docx]

**SUPPORTING INFORMATION**

**Figure S1**. **Diagram of the experimental procedure.**
