# Supplementary material for: Intravital Placenta Imaging Reveals Microcirculatory Dynamics Impact on Sequestration and Phagocytosis of Plasmodium-Infected Erythrocytes
Source: PLoS Pathog. 2013 Jan 31;9(1):e1003154. doi: 10.1371/journal.ppat.1003154 (PMC3561179; doi:10.1371/journal.ppat.1003154)
Supplement: Figure S2 — Parasitized erythrocytes in the periphery. Non-pregnant BALB/c female was infected with P. berghei-ANKA and imaged 1 week after infection. Sequential images of blood circulation inside the popliteal lymph-node show IE (indicated by arrows) travelling at speed over 20 µm/s (velocity was calculated by Fiji imageJ manual tracking plug-in; data not shown) (see Video S10). Red: blood-labeled Dextran-Rhodamine; green; IE. Scale bar: 50 µm. (DOCX) [file ppat.1003154.s002.docx]

**SUPPORTING INFORMATION**

**Figure S2**. **Parasitized erythrocytes in the periphery.**
