# Supplementary material for: Intravital Placenta Imaging Reveals Microcirculatory Dynamics Impact on Sequestration and Phagocytosis of Plasmodium-Infected Erythrocytes
Source: PLoS Pathog. 2013 Jan 31;9(1):e1003154. doi: 10.1371/journal.ppat.1003154 (PMC3561179; doi:10.1371/journal.ppat.1003154)
Supplement: Figure S3 — Deposition of amorfous eosinophilic fibrinoid material in the lumina of maternal blood space (MBS) (A), often associated with necrotic cell debris (B) (arrows). Thrombus formation was also registered (C). H&E sagittal section of P.berghei-infected placentas at G18. FC: fetal capillary; IM: interheamal membrane. (DOCX) [file ppat.1003154.s003.docx]

**SUPPORTING INFORMATION**

**Figure S3.** Deposition of amorfous eosinophilic fibrinoid material
